# Zika virus infects renal proximal tubular epithelial cells with prolonged persistency and cytopathic effects

**DOI:** 10.1038/emi.2017.67

**Published:** 2017-08-23

**Authors:** Jian Chen, Yi-feng Yang, Jun Chen, Xiaohui Zhou, Zhaoguang Dong, Tianyue Chen, Yu Yang, Peng Zou, Biao Jiang, Yunwen Hu, Lu Lu, Xiaoyan Zhang, Jia Liu, Jianqing Xu, Tongyu Zhu

**Affiliations:** 1 Scientific Research Center, Shanghai Public Health Clinical Center, Fudan University, Shanghai 201508, China; 2 Shanghai Institute for Advanced Immunochemical Studies, ShanghaiTech University, Shanghai 201210, China; 3 Department of Ecology and Genetics, Evolutionary Biology Centre, Uppsala University, Uppsala 75236, Sweden; 4 Institutes of Biomedical Sciences, Key Laboratory of Medical Molecular Virology of Ministry of Education/Health, Fudan University, Shanghai 200032, China; 5 Department of Urology, Zhongshan Hospital, Fudan University, Shanghai 200032, China; 6 Shanghai Key Laboratory of Organ Transplantation, Zhongshan Hospital, Fudan University, Shanghai 200032, China

**Keywords:** cytopathic effects, prolonged persistency, renal proximal tubular epithelial cells, urinary system, Zika virus

## Abstract

Zika virus (ZIKV) infection can cause fetal developmental abnormalities and Guillain–Barré syndrome in adults. Although progress has been made in understanding the link between ZIKV infection and microcephaly, the pathology of ZIKV, particularly the viral reservoirs in human, remains poorly understood. Several studies have shown that compared to serum samples, patients’ urine samples often have a longer duration of ZIKV persistency and higher viral load. This finding suggests that an independent viral reservoir may exist in the human urinary system. Despite the clinical observations, the host cells of ZIKV in the human urinary system are poorly characterized. In this study, we demonstrate that ZIKV can infect renal proximal tubular epithelial cells (RPTEpiCs) in immunodeficient mice *in vivo* and in both immortalized and primary human renal proximal tubular epithelial cells (hRPTEpiCs) *in vitro*. Importantly, ZIKV infection in mouse kidneys caused caspase-3-mediated apoptosis of renal cells. Similarly, *in vitro* infection of immortalized and primary hRPTEpiCs resulted in notable cytopathic effects. Consistent with the clinical observations, we found that ZIKV infection can persist with prolonged duration in hRPTEpiCs. RNA-Seq analyses of infected hRPTEpiCs revealed a large number of transcriptional changes in response to ZIKV infection, including type I interferon signaling genes and anti-viral response genes. Our results suggest that hRPTEpiCs are a potential reservoir of ZIKV in the human urinary system, providing a possible explanation for the prolonged persistency of ZIKV in patients’ urine.

## INTRODUCTION

A Zika virus (ZIKV) infection is typically accompanied by mild symptoms that are present in 20% infected individuals.^1^ However, the 2015 ZIKV outbreak in Brazil was associated with microcephaly, a severe developmental abnormality of the fetal brain.^2^ *In vivo* studies of mice^3, 4, 5, 6^ and non-human primates^7^ have established a causal link between ZIKV infection and the birth defects of newborns. *In vitro* studies have identified neural progenitor cells (NPCs) and neural stem cells (NSCs) as the permissive host cells of ZIKV in the central nervous system, providing insights into the mechanism of ZIKV-induced fetal demise.^8, 9, 10, 11^ In addition, ZIKV can infect and cause damage in the human peripheral nervous system, which may lead to Guillain–Barré syndrome (GBS) in adults.^12, 13^

Considerable efforts have been devoted to understanding the viral reservoir of ZIKV in human due to its importance in the prevention and treatment of ZIKV infections. It has been reported that sexual transmission of ZIKV can occur from males to females,^14, 15, 16^ males to males^17^ and, in one suspected case, a female to a male.^18^ ZIKV may also be transmitted from sexual partners without ZIKV-related symptoms.^16^ ZIKV can exist in human semen^19, 20^ and in the genital tract.^21, 22, 23^ In support of these clinical findings, studies using mouse models have shown that ZIKV can infect and replicate in the vagina^24, 25^ and that infection of testes led to testis damage and male infertility.^26, 27^ These studies uncovered a ZIKV reservoir in the reproductive system apart from that in the nervous system. ZIKV has been reported to exist in the urine of adults^28, 29, 30, 31, 32^ and in neonates^29^ as well as in the amniotic fluids (composed primarily of fetal urine) of ZIKV-infected fetuses.^33, 34^ Interestingly, although in most patients ZIKV RNA cannot be detected in the sera after the first week of illness,^35, 36^ their urine samples have detectable levels of ZIKV RNA until at least 2 weeks after the onset of symptoms.^30, 36, 37, 38^ The United States Centers for Disease Control and Prevention (CDC) has recommended the use of urine samples for the non-invasive diagnosis of ZIKV.^39^ Nevertheless, it is still debatable whether urine should be used for the molecular diagnosis of ZIKV.^40, 41, 42^ This uncertainty is, in part, due to the unidentified source of ZIKV in the urine samples. Because urinary ZIKV typically exhibits a higher viral load and longer duration than that in the sera,^32^ the urinary system may present an independent viral reservoir that is different from the bloodstream.

Renal infections caused by ZIKV are largely unknown. However, a previous study showed that ZIKV could infect renal glomerular cells.^43^ In this study, we showed that ZIKV can infect renal tubular cells in an immunodeficient mouse model. In addition, we found that the *in vitro* ZIKV infection of human RPTEpiCs (hRPTEpiCs) resulted in the prolonged persistency of viruses. Both *in vivo* and *in vitro* ZIKV infections resulted in caspase-3-induced cell apoptosis. RNA-Seq analyses of ZIKV-infected hRPTEpiCs revealed a notable transcriptional change of a large set of genes, including type I interferon signaling and anti-viral response genes.

## MATERIALS AND METHODS

### Cell lines and viruses

*Aedes albopictus* C6/36 cells were grown in 30% RPMI-1640 (Gibco-Life Technologies, Carlsbad, CA, USA), 60% Dulbecco’s modified Eagle’s medium (DMEM, Gibco) supplemented with 10% fetal bovine serum (FBS, Gibco) at 28 °C, as previously described.^44^ HK2 cells were cultured in DMEM/F12 (1:1, Gibco) supplemented with 10% FBS, 100 IU/mL of penicillin and 100 μg/mL of streptomycin and were maintained at 37 °C in a fully humidified atmosphere with 5% CO_2_. Primary hRPTEpiCs were purchased from ScienCell (Carlsbad, CA, USA) and maintained on polylysine (ScienCell)-coated plates in Epithelial Cell Medium (EpiCM, ScienCell) supplemented with 2% FBS (ScienCell), 1% epithelial cell growth supplement (EpiCGS, ScienCell) and 1% penicillin/streptomycin solution (P/S, ScienCell). The SZ01 ZIKV stock^45^ was kindly provided by Professor Cheng-Feng Qin at the Department of Virology, State Key Laboratory of Pathogen and Biosecurity, Beijing Institute of Microbiology and Epidemiology. The MR766 ZIKV stock was obtained from ATCC (VR-1838; Manassas, VA, USA).

### Animal experiments

All animal experiments described in this study were approved by Fudan University and ShanghaiTech University. Animal studies were carried out in strict accordance with the Institutional Animal Care and Use Committee approved protocols. Inoculation of animals with ZIKV was performed under anesthesia using chloral hydrate, and all efforts were made to minimize animal suffering.

C57BL/6 mice deficient of type I and II interferon (IFN) receptors (AG6 mice) were purchased from the B&K Universal Group Limited (Shanghai, China) and housed under specific pathogen-free (SPF) conditions at the animal facilities of the Shanghai Public Health Clinical Center, Fudan University (Shanghai, China). The mice were transferred to the Animal Biosafety Level 2 Laboratory before infection. Groups of 4- to 8-week-old mice of both sexes were used for all experiments. All mice were intraperitoneally (i.p.) inoculated with 10^5^ plaque-forming unit (PFU) of ZIKV in a 100 μL volume. Kidneys were harvested from both mock and infected mice at 7 days post infection and were immediately fixed for 16 h in 10% neutral buffered formalin (NBF). Fixed kidneys were mounted and sectioned into 10-μm slices for RNA *in situ* hybridization (ISH) and hematoxylin and eosin (H&E) staining.

RNA ISH was performed using a RNAscope 2.5 HD Reagent Kit-BROWN (Advanced Cell Diagnostics, Newark, CA, USA) according to the manufacturer’s instructions. NBF-fixed tissue slides were hydrated and then immersed successively in 200 mL of 50% ethanol, 200 mL of 70% ethanol and 400 mL of 100% ethanol for 5 min each at room temperature. ZIKV genomic RNA was detected using an RNA probe from Advanced Cell Diagnostics (Cat No. 467771). Hybridization signals were visualized by chromogenic reactions using DAB chromogen, followed by counterstain with Gill’s hematoxylin. RNA ISH and H&E stain samples were photographed using a Tissue FAXS 200 flow-type quantitative tissue analyzer (TissueGnostics GmbH, Vienna, Austria).

### Quantification of ZIKV

The ZIKV viral RNA in the supernatant of medium was quantified by RT-qPCR. Viral RNA was extracted using a QIAamp viral RNA mini kit (Qiagen, Valencia, CA, USA) according to the manufacturer’s instructions. One microgram of RNA was transcribed into cDNA using random primers using Moloney murine leukemia virus (M-MLV) reverse transcriptase (Promega, Charbonnieres, France) and the cDNA product was amplified by RT-qPCR using primers SZ01-1F (CAA GGA GTG GGA AGC GGA G) and SZ01-1R (CCA TGT GAT GTC ACC TGC TCT) and the T7 RiboMAX Express RNAi System (Promega) on an Applied Biosystems 7300 real-time PCR cycler (Applied Biosystems, Carlsbad, CA, USA). qPCR data were analyzed using SDS software from Applied Biosystems and the viral RNA was quantified by comparing each sample’s threshold cycle (CT) value with a ZIKV RNA standard curve. The TCID_50_ of each collected supernatant was determined by titrating HK2 cells in a 96-well plate.

### Immunofluorescence and immunohistochemistry experiments

For immunofluorescence (IF) experiments, frozen kidney sections (10 μm) and hRPTEpiC slices were fixed with ice-cold acetone for 10 min. The sections were washed with PBS for four times and blocked with blocking buffer (1% BSA and 2% donkey serum diluted in PBS) at room temperature for 30 min. Primary antibodies were diluted in blocking buffer and incubated overnight at 4 °C; the primary antibodies used were anti-flavivirus envelope protein antibody (1: 200, clone D1-4G2-4-15, Millipore, Darmstadt, Germany), rabbit anti-Vimentin (1:200, #5741, Cell Signaling Technology, CST, Danvers, MA, USA) and rabbit anti-cleaved caspase-3 (1:400, #9661, CST). The secondary antibodies used were Alexa Fluor 488 donkey anti-rabbit IgG H&L (1:1 000, ab150073, Abcam) and Alexa Fluor 568 donkey anti-mouse IgG (H+L) (1:1 000, ab175472, Abcam, Cambridge, UK).

For immunohistochemical (IHC) staining, frozen kidney sections (10 μm) were treated as described above. Primary antibodies were diluted in blocking buffer and incubated overnight at 4 °C; the primary antibodies used were rat anti-CD4 (1:200, ab25475, Abcam), rat anti-CD8 (1:400, ab22378, Abcam), rat anti-neutrophile (1:200, ab2557, Abcam) and rat anti-monocyte chemoattractant protein-1 (MCP-1; 1:200, ab8101, Abcam). After rinsing with PBS, the primary antibodies were subsequently detected with a rabbit anti-rat IgG H&L (HRP) secondary antibody. Sections were lightly counterstained with Mayer’s hematoxylin. Images were taken using a Tissue FAXS 200 flow-type tissue quantitative analyzer (TissueGnostics GmbH, Vienna, Austria).

### Statistics

For RT-qPCR and TCID_50_ quantification, five biological replicates were performed, and the results are displayed as the mean±sd. Unless otherwise noted, statistical analyses were performed using two-tailed Student’s *t*-test.

### Whole-transcriptome (RNA-Seq) analysis

Mock and infected hRPTEpiCs (three replicates in each group) were harvested at 48 h post infection. Whole-transcriptome sequencing was performed by Genergy Biotechnology Inc. (Shanghai, China). RNA-Seq short reads were aligned to the human genome (GRCh38) using GSNAP^46^ with a maximum of two mismatches. On average, ~23.7 million reads across all samples were aligned to the reference, which accounted for 95.9% of total reads. Gene expression was determined as number of short reads that fully/partially aligned to the annotated gene model using HTseq.^47^ Expressed genes were defined as those genes having more than 10 total mapped reads in all samples with at least two of three replicates having more than two reads. In total, 17 699 genes met the criteria and were defined as expressed in both mock and ZIKV-infected libraries. Differential expressed genes (DEGs) were identified using the R Bioconductor package ‘edgeR’.^48^ Trimmed mean of M-values normalization was applied to account for differences in library size among samples. Read counts were fitted into generalized linear model with negative binomial distribution and the effect of viral infection was evaluated by a likelihood ratio test. *P*-values were adjusted for multiple tests using false discovery rate.^49^ Significant DEGs were identified with a FDR ≤0.05 and a log_2_(fold change) ≥1. Gene ontology enrichment analysis was performed using GOrilla^50^ by comparing the up/downregulated DEGs to a list of all expressed genes. Significant GO terms with FDR ≤0.05 were reported.

## RESULTS

### Viral production and ZIKV infection in a mouse model

Although ZIKV can be detected in patients’ urine sample, no renal abnormalities or biopsies in humans have been reported to date. To identify the host cells of ZIKV in kidneys, we chose to use type I and II IFN receptor-deficient mice (AG6) to model the *in vivo* renal infection of ZIKV. SZ01, an Asian-lineage ZIKV,^45^ was produced in C6/36 cells in high quantity and PFU. Buffered saline or 1 × 10^5^ PFU of SZ01 ZIKV was inoculated into AG6 mice via i.p. injection. Kidneys from both the mock and ZIKV groups were harvested and sectioned at day 7 post infection. No bleeding or anatomic abnormalities of kidneys were observed.

### ISH, IHC and IF studies of infected kidneys

**Figure 1:**
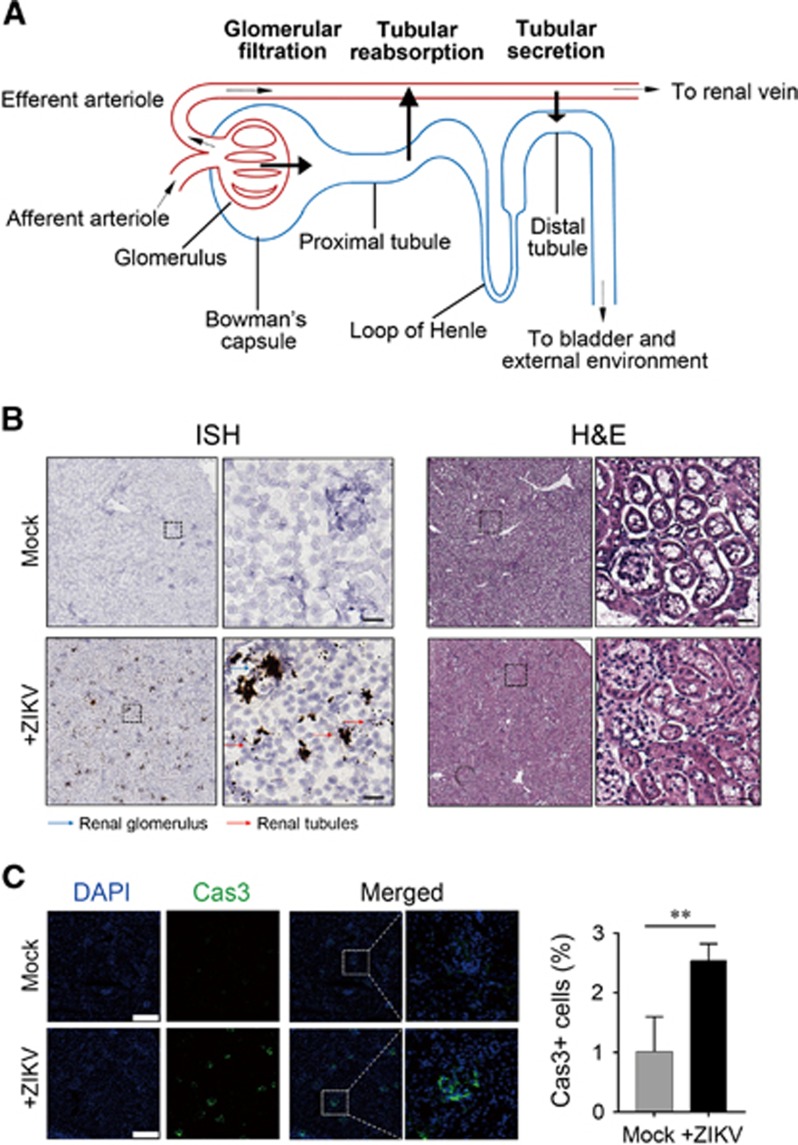
Renal infection of Zika virus (ZIKV) in AG6 mice. (**A**) Components of the nephron. (**B**) Detection of ZIKV RNA in mouse kidneys. Left panel, *in situ* hybridization (ISH) using a ZIKV-specific probe. Blue and red arrows indicate renal glomerular and tubular cells, respectively. Hybridization signals of ZIKV RNA were developed by a chromogenic reaction and are observed as black particles. Right panel, hematoxylin and eosin (H&E) staining. Scale bar, 50 μm. (**C**) Immunofluorescence staining of caspase-3 in ZIKV-infected renal tissues of AG6 mice. Left panel, representative images. Scale bar, 200 μm. Right panel, statistical analyses of caspase-3-positive cells. **Significant difference between mock and infected cells (*P*<0.01; Student’s *t*-test).

The nephron is the basic unit of the kidney and is composed of a renal corpuscle and a tubule (Figure 1A). The renal corpuscle is the initial filtering component of the kidney, consisting of glomerulus and Bowman’s capsule. According to the relative positions, renal tubules are categorized into the proximal tubule, the loop of Henle and the distal tubule. The proximal tubule has a brush border, or so-called microvilli, and thus has a larger lumen than the PCT lumen. Most of the reabsorption and secretion occurs in proximal tubule.

In our studies, the ZIKV RNA in infected mouse kidneys were detected by ISH using a ZIKV-specific probe. We found that ZIKV RNA was detected in both the renal glomerulus and tubule (Figure 1B, left panel). It appeared that ZIKV signals clustered in the renal glomerulus, whereas the renal tubule displayed a lesser degree of ZIKV signals. This result suggests that ZIKV may penetrate the glomeruli and then into tubules during renal filtration. H&E staining revealed ZIKV-induced swelling of kidneys (Figure 1B, right panel). IF staining of infected renal cells using an anti-caspase-3 antibody indicated ZIKV-induced apoptosis (Figure 1C). IHC staining indicated that infiltrating CD8^+^ cells were significantly increased in ZIKV-infected kidneys, whereas no difference in infiltrating neutrophils or MCP-1 expression was observed between the control and ZIKV-infected kidneys (Supplementary Figure S1). Taken together, our results suggest that mouse renal tubular cells are susceptible *in vivo* host cells of ZIKV.

### ZIKV infection in the immortalized hRPTEpiC line HK2

**Figure 2:**
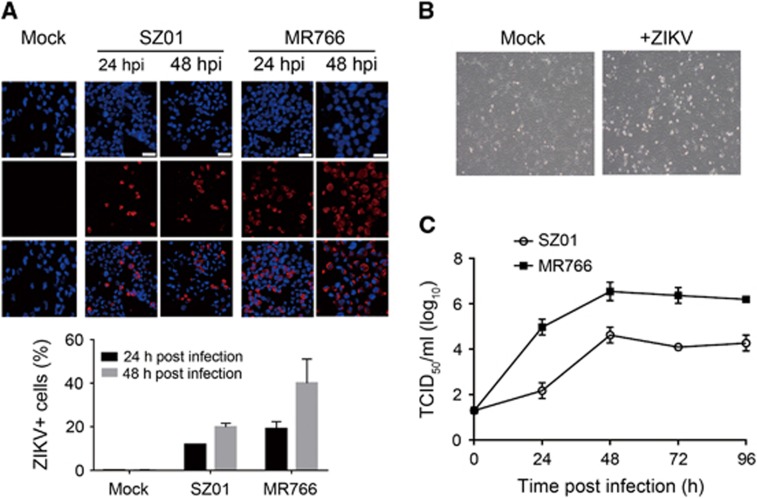
ZIKV infection in the HK2 cell line. (**A**) Immunofluorescence staining of HK2 cells infected with SZ01 or MR766 ZIKV using an anti-ZIKV envelope protein antibody. Upper panel, representative images. Scale bar, 20 μm. Lower panel, quantification of infected cells. (**B**) Light microscopy images showing SZ01 ZIKV-induced CPEs. (**C**) Production of infectious SZ01 and MR766 ZIKV in HK2 cells. Cytopathogenic effects, CPEs.

We next sought to assess ZIKV infection in human renal tubular cells. We first evaluated ZIKV infection in an immortalized hRPTEpiC line, HK2, which was derived from a normal adult human kidney.^51^ To avoid bias of a single virus strain, we used both SZ01, an Asian-lineage ZIKV, and MR766, an African-lineage ZIKV (ATCC VR-1838), for our study. IF staining using an anti-ZIKV envelop protein (ZIKVE) antibody showed that both SZ01 and MR766 could efficiently infect HK2 cells at 24 and 48 h post inoculation (Figure 2A). The use of MR766 ZIKV appeared to result in higher infection rate than SZ01 ZIKV. In addition, ZIKV infection induced notable cytopathogenic effects (CPEs) in HK2 cells at 48 h post infection (Figure 2B). To determine the production of infectious viral particles, we analyzed the TCID_50_ of SZ01 and MR766 ZIKV from infected HK2 cells over a period of 96 h. The results indicated that HK2 could produce infectious SZ01 and MR766 particles in a time-dependent manner. In addition, with the same initial viral dose, HK2 produced more infectious MR766 particles than SZ01 at various time points.

### ZIKV infection in primary hRPTEpiCs

**Figure 3:**
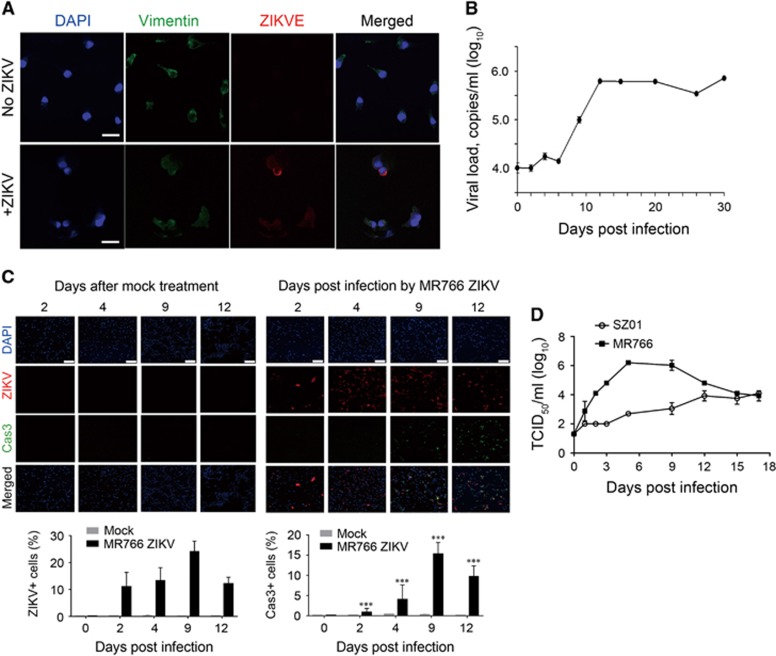
ZIKV infection in primary hRPTEpiCs. (**A**) Immunofluorescence staining of primary hRPTEpiCs infected with SZ01 ZIKV at a MOI of 2.5 for 96 h. Scale bar, 30 μm. (**B**) Long-term persistence of SZ01 ZIKV in primary hRPTEpiCs. Viral copies in the supernatant of medium were determined by RT-qPCR using ZIKV standard samples. Values are shown as the mean±standard deviation (sd; *n*=5). (**C**) MR766 ZIKV-induced apoptosis in primary hRPTEpiCs. Upper panel, representative immunofluorescence images. Scale bar, 200 μm. Lower panel, quantification of ZIKV-positive and caspase-3-positive cells. ***Significant difference between mock and infected cells (*P*<0.001; Student’s *t*-test). (**D**) Production of infectious SZ01 and MR766 ZIKV particles in primary hRPTEpiCs.

Next, we explored ZIKV infection in primary hRPTEpiCs. ZIKV-infected primary hRPTEpiCs were stained with a ZIKV antibody and an antibody against vimentin, a marker of hRPTEpiCs. We found that hRPTEpiCs could be efficiently infected by ZIKV (Figure 3A), which is in line with the results observed in the HK2 cell line. We next assessed viral replication in hRPTEpiCs by quantifying ZIKV RNA in the supernatant of medium using RT-qPCR. ZIKV was found to persist in hRPTEpiCs for more than 30 days after infection (Figure 3B), which is consistent with the prolonged persistency of ZIKV in patients’ urine samples. Moreover, the MR766 ZIKV infection induced CPEs in hRPTEpiCs over a course of 12 days, as evidenced by the increased cell apoptosis (Figure 3C). By contrast, the mock treatment led to little or no cell apoptosis. In addition, Asian-lineage ZIKV SZ01 induced caspase-3-mediated apoptosis in hRPTEpiCs (Supplementary Figure S2). It was noted that SZ01 ZIKV only induced a modest degree of apoptosis after an extended period of elongated infection, whereas MR766 ZIKV induced a notably higher level of apoptosis at all examined time points. Similar to the results obtained with the HK2 cell line, primary hRPTEpiCs could produce both SZ01 and MR766 infectious viral particles over a course of 17 days. It was also noted that primary hRPTEpiCs produced more infectious MR766 than SZ01 at various time points (Figure 3D). Collectively, these results suggest that hRPTEpiCs are a permissive cell host of ZIKV and that MR766 has higher fitness in hRPTEpiCs than SZ01.

### RNA-Seq analysis of ZIKV-infected hRPTEpiCs

**Figure 4:**
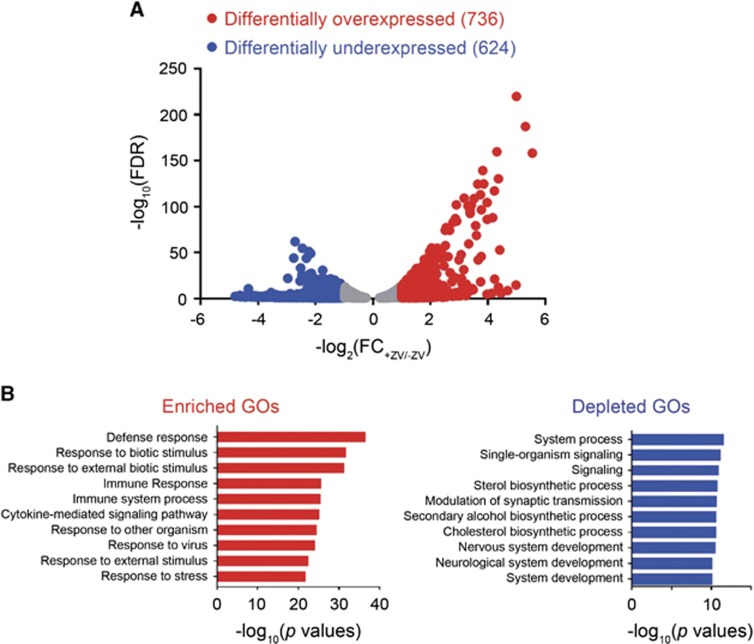
RNA-Seq analysis of mock and ZIKV-infected primary hRPTEpiCs at 48 h post infection. (**A**) Volcano plot of genes differentially expressed in mock and ZIKV-infected hRPTEpiCs. (**B**) Genes with differential expression between mock and infected hRPTEpiCs were subjected to gene ontology (GO) analyses. The top 10 most significantly upregulated and downregulated biological processes are shown in red and blue, respectively.

To understand the cellular response of hRPTEpiCs to ZIKV infection, we performed RNA-Seq analysis on mock and ZIKV-infected primary hRPTEpiCs at 48 h post infection. Analysis of the whole transcriptome revealed a large set of dysregulated genes in ZIKV-infected hRPTEpiCs, including 726 upregulated genes and 624 downregulated genes (Figure 4A). The top 10 up- and downregulated genes exhibited up to a 40- or 20-fold change of mRNA expression, respectively (Supplementary Tables S1 and S2). Gene ontology (GO) analysis uncovered a variety of perturbed biological processes, including upregulated immune response and cytokine-mediated signaling pathways (Figure 4B). The downregulated biological processes are composed primarily of developmental GO terms. Analyses of differentially expressed genes (DEGs) showed that ZIKV infection stimulated the upregulation of a variety of type I IFN signaling, anti-viral responses and inflammatory genes (Supplementary Figure S3).

## DISCUSSION

First discovered in 1947 in the Zika forest of Uganda, ZIKV initially gained little notice as a threat to public health due to the benign symptoms of infection. However, the ZIKV outbreak in Brazil in 2015 was found to be associated with a notable rise of neonate microcephaly, a medical condition characterized by developmental abnormalities of fetal brains.^2^ Clinical and laboratory studies have confirmed the causal link between ZIKV infection and fetal microcephaly.^52^ A recent study has suggested that the primary targets of ZIKV in the central nervous system are astrocytes,^53^ although neural progenitor cells^8^ and neural stem cells^11^ can also be infected. However, the transmission pathway of ZIKV within the central nervous system, as well as the physiological relevance of the prolonged persistency of ZIKV in human fetal neural progenitors,^9^ remains unclear. In addition, ZIKV can damage the peripheral nervous system, leading to a progressive neurological disease known as GBS that causes severe muscle weakness in adults.^13^ The mechanism of ZIKV-induced GBS is largely unknown. In addition to the nervous system, recent studies have highlighted the presence of a viral reservoir of ZIKV in the reproductive system.^24, 27^

Despite progress, a long-standing unexplored question is why ZIKV persists longer and has a higher load in urine than in serum. It is most likely that an independent viral reservoir exists in the urinary system. A recent study has shown that ZIKV can infect glomerular podocytes and renal glomerular endothelial and mesangial cells.^43^ In this study, we showed that inoculation with ZIKV in mice deficient of type I and II IFN receptors led to viral replication in both renal glomeruli and tubules. In addition, renal glomeruli presented a higher density of ZIKV signals than renal tubules. This result suggested a possible mechanism of the penetration of plasma ZIKV to renal tubular cells through filtration of renal glomeruli. We also found that ZIKV exhibited long-term persistence in primary hRPTEpiCs. Moreover, two strains of ZIKV, SZ01 and MR766, could both replicate in immortalized and primary hRPTEpiCs as infectious viral particles. These results together suggest that hRPTEpiCs in kidneys may serve as a viral reservoir for persistent viral replication. In addition, both *in vitro* and *in vivo* ZIKV infections induced notable caspase-3-mediated cell apoptosis. These observations suggest caution for ZIKV-associated renal abnormalities. It would therefore be very interesting to investigate ZIKV-induced renal pathogenesis in humans in future studies.

We also noted that different African and Asian ZIKV strains exhibited notably different infectious profiles. Compared with Asian-lineage ZIKV SZ01, African ZIKV MR766 produced a higher level of infectious viral particles in both immortalized and primary hRPTEpiCs (Figures 2C and 3D). In addition, MR766 induced higher degree of cell apoptosis than SZ01 (Figure 3C). Previous studies have shown that African ZIKV could lead to a higher infection rate and virus-induced cell death *in vitro*^54^ and *in vivo*^55^ compared with those treated with Asian ZIKV. Our current findings are in line with previous results and indicate that African- and Asian-lineage ZIKV have strain-specific pathogenesis profiles that should be taken into account in future studies.

We noted that our RNA-Seq results were different from those obtained from ZIKV-infected human neural progenitor cells, whereby the majority of the most significantly dysregulated GO terms were cell death or cell cycle-related biological processes.^8^ This discrepancy could be due to the cell type-specific response to ZIKV infection. In addition, RNA-Seq analyses suggested that ZIKV did not inhibit the anti-viral response of hRPTEpiCs as was seen in other cell types.^56^ This result is likely attributable to the different innate immune response mechanisms in various cell types.

Another important implication of our study is that it provides experimental support for the CDC-approved diagnostic method of ZIKV. The lack of laboratory studies regarding the renal infection of ZIKV, especially the unclear origin of prolonged ZIKV persistency in urine, raised concerns of whether urine samples should be used for the accurate diagnosis of ZIKV.^40, 42^ Our study showed that ZIKV can infect RPTEpiCs both *in vitro* and *in vivo*, thus rationalizing the use of urine samples for non-invasive ZIKV diagnosis.
