# Supplementary material for: Zika virus infects renal proximal tubular epithelial cells with prolonged persistency and cytopathic effects
Source: Emerg Microbes Infect. 2017 Aug 23;6(8):e77–. doi: 10.1038/emi.2017.67 (PMC5583673; doi:10.1038/emi.2017.67)
Supplement: Supplementary Figure S1 [file emi201767x1.pdf]

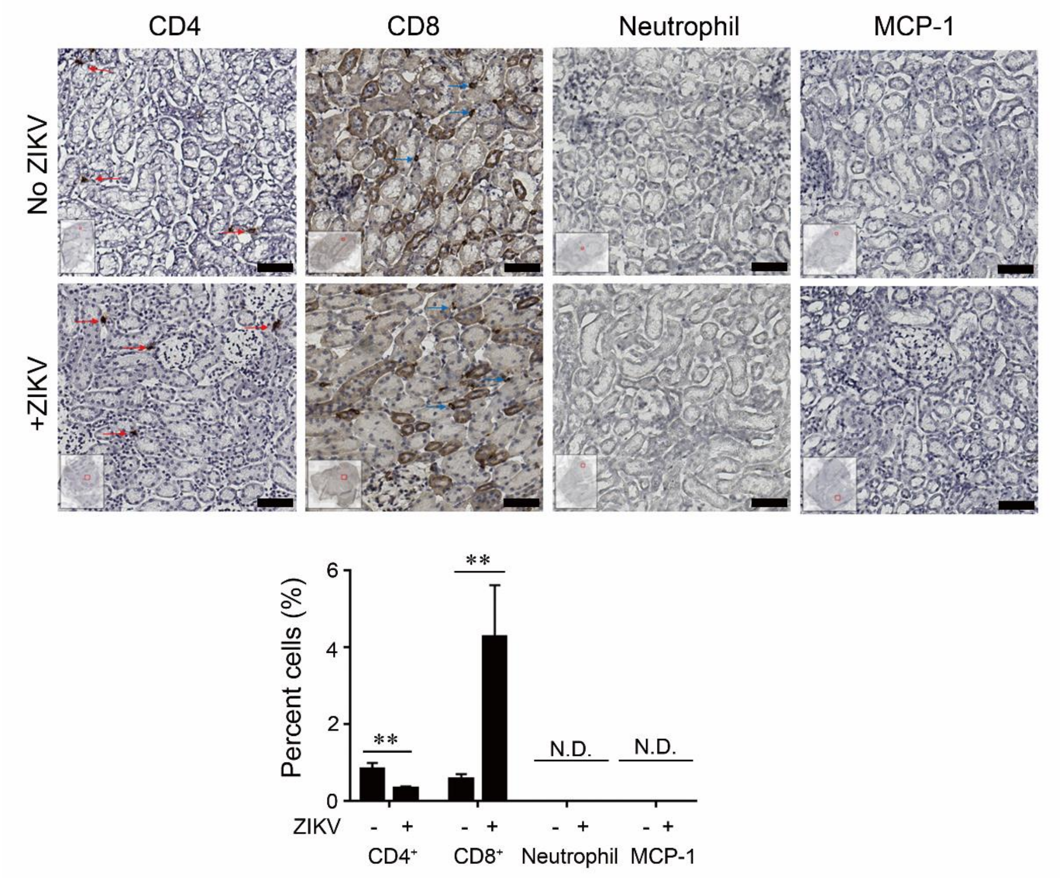

Supplemental Figure 1. Immunohistochemical staining of kidneys in mock and ZIKV-infected mice. MCP-1, monocyte chemoattractant protein-1. Upper panel, representative images. Red and blue arrows donate infiltrated CD4$^+$ and CD8$^+$ T cells, respectively. Scale bar, 50 μm. Lower panel, statistical analyses. **, significant difference between mock and infected groups (p< 0.01, Student's t test). N.D., not detected.
