# Supplementary material for: Zika virus infects renal proximal tubular epithelial cells with prolonged persistency and cytopathic effects
Source: Emerg Microbes Infect. 2017 Aug 23;6(8):e77–. doi: 10.1038/emi.2017.67 (PMC5583673; doi:10.1038/emi.2017.67)
Supplement: Supplementary Figure S2 [file emi201767x2.pdf]

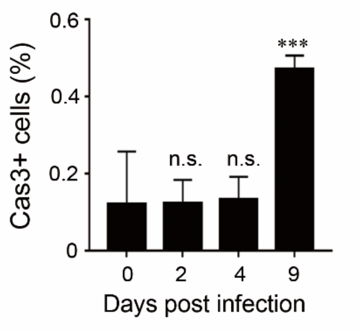

Supplemental Figure 2. SZ01 ZIKV-induced apoptosis in primary hRPTEpiCs. ***, significantly different from day 0 group (*p*< 0.001, Student's *t* test). n.s., not significantly different from day 0 group.
