# Supplementary material for: Zika virus infects renal proximal tubular epithelial cells with prolonged persistency and cytopathic effects
Source: Emerg Microbes Infect. 2017 Aug 23;6(8):e77–. doi: 10.1038/emi.2017.67 (PMC5583673; doi:10.1038/emi.2017.67)
Supplement: Supplementary Figure S3 [file emi201767x3.pdf]

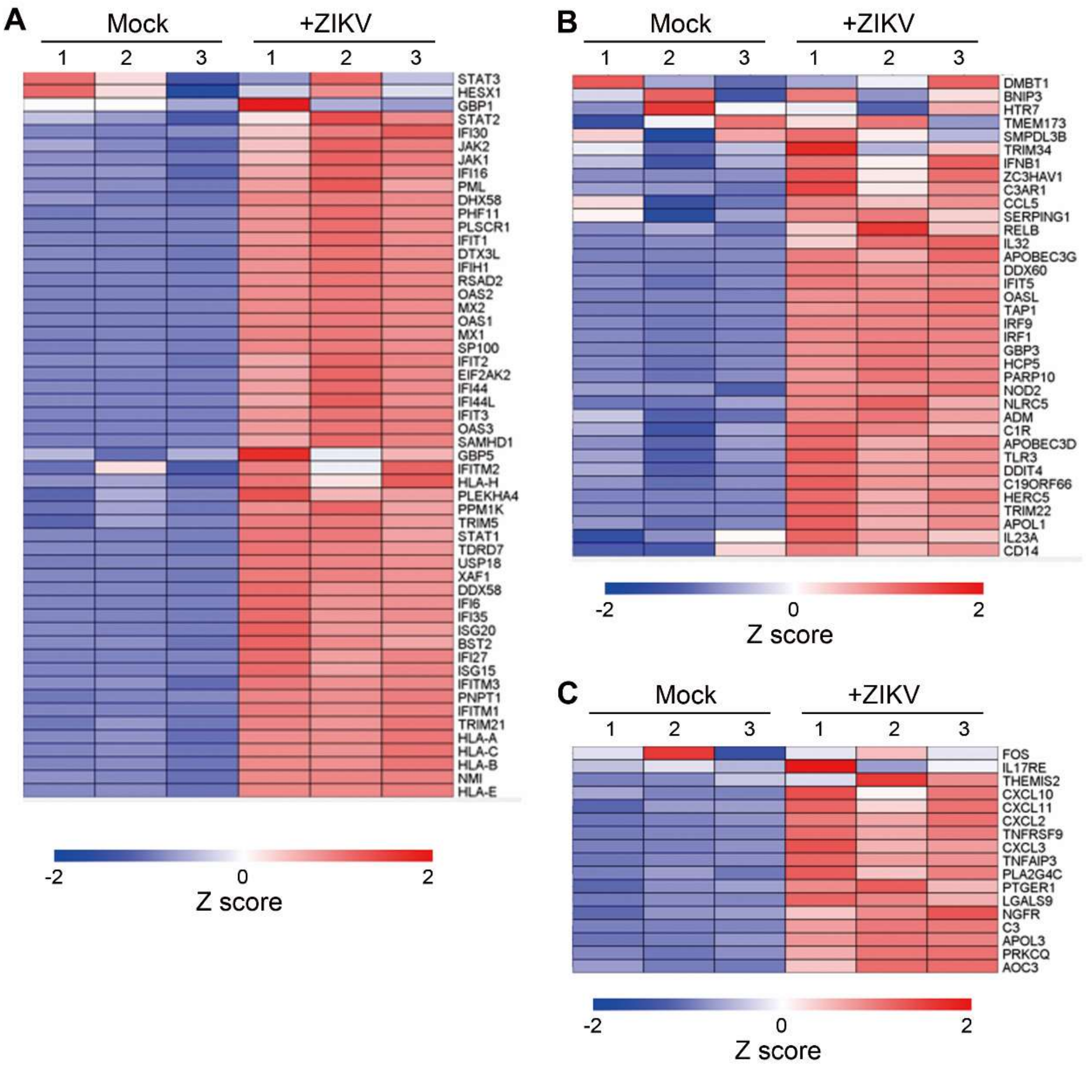

Supplementary Figure 3. ZIKV-induced up-regulation of type I IFN signaling (A), anti-viral responsive (B) and inflammatory genes (C). Differential gene expression is displayed as Z-score.
