# Supplementary material for: Zika virus infects renal proximal tubular epithelial cells with prolonged persistency and cytopathic effects
Source: Emerg Microbes Infect. 2017 Aug 23;6(8):e77–. doi: 10.1038/emi.2017.67 (PMC5583673; doi:10.1038/emi.2017.67)
Supplement: Supplementary Table S1 [file emi201767x4.pdf]

Supplementary Table 1. Top 30 up-regulated genes upon ZIKV infection.

**Gene symbol**	**Description**	**log_2FC**	***P* value**
KLHDC7B	kelch domain containing 7B	5.54939	1.20E-162
IFI27	interferon alpha inducible protein 27	5.30723	8.99E-192
MX2	MX dynamin like GTPase 2	4.99477	6.73E-225
VGF	VGF nerve growth factor inducible	4.98097	2.55E-17
LCN2	lipocalin 2	4.68728	5.55E-11
HSH2D	hematopoietic SH2 domain containing	4.45578	7.96E-08
LAMP3	lysosomal associated membrane protein 3	4.41970	4.29E-56
RSAD2	radical S-adenosyl methionine domain containing 2	4.37088	1.39E-134
RAB39B	RAB39B, member RAS oncogene family	4.36688	6.70E-15
BIRC3	baculoviral IAP repeat containing 3	4.31874	3.17E-164
FBXO39	F-box protein 39	4.23834	6.80E-24
IFI44L	interferon induced protein 44 like	4.22714	3.30E-121
OASL	2'-5'-oligoadenylate synthetase like	4.16907	1.39E-91
SMTNL1	smoothelin like 1	4.13638	1.10E-07
ODF3B	outer dense fiber of sperm tails 3B	3.97927	9.15E-90
ISG15	ISG15 ubiquitin-like modifier	3.97379	1.95E-108
ZBP1	Z-DNA binding protein 1	3.97177	1.25E-06
MX1	MX dynamin like GTPase 1	3.85401	6.59E-129
IFI6	interferon alpha inducible protein 6	3.82001	1.59E-143
HERC5	HECT and RLD domain containing E3 ubiquitin protein ligase 5	3.77968	3.25E-100
ITPKA	inositol-trisphosphate 3-kinase A	3.76800	4.36E-49
PCDH1	protocadherin 1	3.74880	6.71E-21
CMPK2	cytidine/uridine monophosphate kinase 2	3.74050	5.40E-117
SYT7	synaptotagmin 7	3.66082	4.43E-45
TYMP	thymidine phosphorylase	3.65178	1.57E-128
C3	complement component 3	3.60419	4.17E-72
SLC15A3	solute carrier family 15 member 3	3.57692	7.07E-83
H1F0	H1 histone family member 0	3.52118	7.82E-113
STK32A	serine/threonine kinase 32A	3.48820	9.46E-12
RPLP0P2	ribosomal protein lateral stalk subunit P0 pseudogene 2	3.45723	2.49E-13

Note: only genes with significant differential expression ($p<0.05$) are listed.
