# Supplementary material for: Zika virus infects renal proximal tubular epithelial cells with prolonged persistency and cytopathic effects
Source: Emerg Microbes Infect. 2017 Aug 23;6(8):e77–. doi: 10.1038/emi.2017.67 (PMC5583673; doi:10.1038/emi.2017.67)
Supplement: Supplementary Table S2 [file emi201767x5.pdf]

Supplementary Table 2. Top 30 down-regulated genes upon ZIKV infection.

Gene symbol	Description	log_2FC	*P* value
GRIN2A	glutamate ionotropic receptor NMDA type subunit 2A	-4.79746	0.00058
GABRG3	gamma-aminobutyric acid type A receptor gamma3 subunit	-4.67209	0.00021
TMEM132C	transmembrane protein 132C	-4.63318	0.00173
CDH18	cadherin 18	-4.62527	0.00004
KCNH5	potassium voltage-gated channel subfamily H member 5	-4.61708	0.00141
HTR2C	5-hydroxytryptamine receptor 2C	-4.56975	0.00070
RXFP1	relaxin/insulin like family peptide receptor 1	-4.49232	0.00116
DSCAM	DS cell adhesion molecule	-4.48518	0.00020
SHISA6	shisa family member 6	-4.47911	0.00068
TRDN	triadin	-4.47033	0.00259
ADCY2	adenylate cyclase 2 (brain)	-4.44641	0.00032
RBFOX1	RNA binding protein, fox-1 homolog 1	-4.43111	0.00006
NRXN1	neurexin 1	-4.42025	0.00024
POU6F2	POU class 6 homeobox 2	-4.41629	0.00070
KHDRBS2	KH RNA binding domain containing, signal transduction associated 2	-4.39610	0.00122
CCDC26	CCDC26 long non-coding RNA	-4.38129	0.00223
LRRTM4	leucine rich repeat transmembrane neuronal 4	-4.36334	0.00013
MIR4300HG	MIR4300 Host Gene	-4.34499	0.00044
LMOD1	leiomodin 1	-4.33658	0.00000
LOC102724623	Uncharacterized	-4.32051	0.00030
RNF219-AS1	RNF219 antisense RNA 1	-4.31415	0.00038
NDST4	N-deacetylase/N-sulfotransferase 4	-4.31229	0.00155
ZNF804B	zinc finger protein 804B	-4.30261	0.00041
ANKFN1	ankyrin repeat and fibronectin type III domain containing 1	-4.28082	0.00035
NXPH1	neurexophilin 1	-4.26383	0.00177
FSTL5	follistatin like 5	-4.26258	0.00027
PCDH11X	protocadherin 11 X-linked	-4.25328	0.00024
LY86-AS1	LY86 antisense RNA 1	-4.23944	0.00082
LINC01483	long intergenic non-protein coding RNA 1483	-4.23537	0.00148
RIT2	Ras like without CAAX 2	-4.23054	0.00109

Note: only genes with significant differential expression ($p<0.05$) are listed.
